# Correction: Generation of stress fibers through myosin-driven reorganization of the actin cortex

**DOI:** 10.7554/eLife.79304

**Published:** 2022-04-20

**Authors:** Jaakko I Lehtimäki, Eeva Kaisa Rajakylä, Sari Tojkander, Pekka Lappalainen

**Keywords:** None

Lehtimäki JI, Rajakylä EK, Tojkander S, Lappalainen P. 2021. Generation of stress fibers through myosin-driven reorganization of the actin cortex. *eLife* **10**:e60710. doi: 10.7554/elife.60710.

Published 28 January 2021

While making an inventory of our siRNA and antibody collections, we identified an error in Fig. 4, supplement 1 (panels C and D) of the publication. We discovered that, instead of silencing mDia1 and mDia2, we depleted mDia1 and mDia3 in experiments shown in the figure. The origin of this error is the nomenclature of mammalian diaphanous family formins (DIAPH2 gene encodes mDia3 formin, and DIAPH3 gene encodes mDia2 formin). We have now also preformed mDia2 silencing and quantified the number of cortical stress fibers in these cells. Our analysis revealed that mDia2 depletion does not markedly affect the numbers of cortical stress fibers. Thus, the earlier error does not affect any of the conclusions of the manuscript, and our study provides evidence that mDia1, mDia2, and mDia3 formins are not necessary for cortical stress fiber assembly.

We regret this error and have corrected the manuscript accordingly.

The corrected Figure 4 - Figure supplement 1 is shown here:

**Figure 1:**
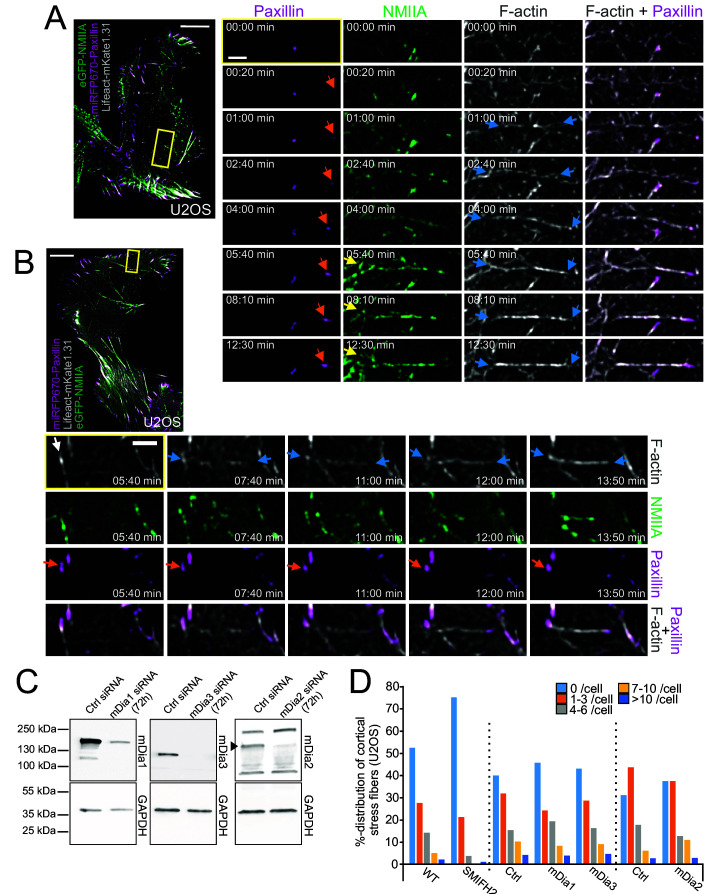


The original Figure 4 - Figure supplement 1 is shown here for reference:

**Figure 2:**
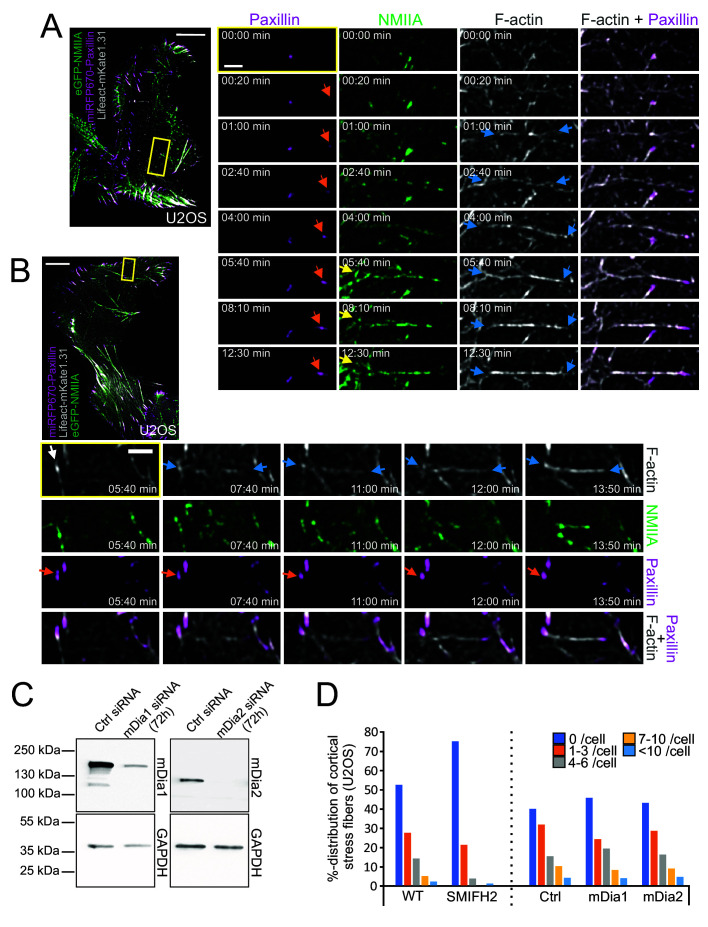


The original figure legend for Figure 4 - figure supplement 1 has changed slightly. Also, Key resources table, Reagents and TIRF microscopy sections in Materials and Methods were edited accordingly (changes are underlined).

Figure legend:

(**C**) Depletion of Dia1 or Dia2 by siRNAs from U2OS cells as detected by western blot. GAPDH was probed to assess equal sample loading. (**D**) Effects of formin inhibitor SMIFH2 (left) and Dia1 and Dia2 depletions (right) on cortical stress fibers as examined by blind analysis of cortical stress fiber numbers (percentual distribution of indicated categories) from tile-scan TIRF data sets.

N = WT 209, Ctrl siRNA 232, mDia1 163, mDia2 138, and SMIFH2 229 U2OS cells analyzed.

To:

(**C**) Depletion of mDia1, mDia3 and mDia2 by siRNAs from U2OS cells as detected by western blot. GAPDH was probed to assess equal sample loading. Black arrowhead points to the mDia2 band with correct MW. (**D**) Effects of formin inhibitor SMIFH2 (left) and mDia1, mDia3 and mDia2 depletions (right) on cortical stress fibers as examined by blind analysis of cortical stress fiber numbers (percentual distribution of indicated categories) from tile-scan TIRF datasets.

N = WT 209, SMIFH2 229, Ctrl siRNA 232 (for mDia1 and mDia3 depletion), mDia1 163, mDia3 138, mDia2 113, and Ctrl siRNA (for mDia2 depletion) 120 U2OS cells analyzed.

**Table:** 

Transfected construct (human)	SMARTpoolON-TARGETplus siRNA to mDia2	Dharmacon	Cat# L-018997-00-0005	Transfected using GeneSilencer
Antibody	Anti- human mDia2 (rabbit polyclonal)	Proteintech	Cat# 14342–1-AP, RRID:AB_2092930	1: 500 (WB)
Antibody	anti- human mDia3 (rabbit polyclonal)	Sigma Aldrich	Cat# HPA005647, RRID:AB_1078657	1:1,000 (WB)

Reagents:

Monoclonal rabbit ab against human mDia1 (Abcam, ab129167) and polyclonal rabbit ab raised against human mDia2 (Sigma Aldrich, HPA005647) were used in 1:1,000 dilution in 5% milk TBS-Tween20 (0.05%) to detect siRNA depletion efficiency.

To:

Monoclonal rabbit ab against human mDia1 (Abcam, ab129167) and polyclonal rabbit abs raised against human mDia2 (Proteintech, 14342–1-AP) and human mDia3 (Sigma Aldrich, HPA005647) were used in 1:500 and 1:1,000 dilution, respectively, in 5% milk TBS-Tween20 (0.05%) to detect siRNA depletion efficiency.

And

To study the role of mDia1 and mDia2 formins in the process, we treated U2OS cells for 72 hr with specific siRNAs (20 µM of SMARTpool ON-TARGETplus, L-010347-00-0005, and L-012029-00-0005, respectively, Dharmacon)

To:

To study the role of mDia, mDia2 and mDia3 formins in the process, we treated U2OS cells for 72 hours with specific siRNAs (20 µM of SMARTpool ON-TARGETplus, L-010347-00-0005, L-018997-00-0005 and L-012029-00-0005 respectively, Dharmacon)

We have updated the TIRF microscopy section on image acquisition of the mDia2 and ctrl siRNA samples by adding a following paragraph:

Tile-scans of mDia2 siRNA- and respective ctrl siRNA- treated samples were performed with Ring-TIRF module of Deltavision OMX SR, as for live-cell TIRF experiments but with following exceptions: imaging was performed at RT, using 1.518 RI immersion oil and 405, 488 and 640 nm diode lasers. 5 × 5 FOVs (1024 × 1024) including 10% overlap, were captured manually, followed by moving to a new area, at least over six times the FOV to another direction, at random on the imaging dish.

Finally, we have updated the ‘Results’ section of the main text:

However, subsequent siRNA experiments on Dia1 and Dia2, which are two formins that were linked to assembly of dorsal stress fibers and transverse arcs, did not result in significant reduction in the number of cortical stress fibers (Figure 4—figure supplement 1C and D). These results suggest that Dia1 and Dia2 are either not critical for generation of cortical stress fibers or that they are functionally redundant with each other or with some other formins expressed in U2OS cells.

To:

However, subsequent siRNA experiments on mDia1, mDia2 and mDia3, from which mDia1-2 have been linked to assembly of dorsal stress fibers and transverse arcs, did not result in significant reduction in the number of cortical stress fibers (Figure 4 – figure supplement 1C-D). These results suggest that mDia1-3 are either not critical for generation of cortical stress fibers, or that they are functionally redundant with each other or with some other formins expressed in U2OS cells.

The article has been corrected accordingly.

